# The Amsterdam Sexual Abuse Case (ASAC)-study in day care centers: longitudinal effects of sexual abuse on infants and very young children and their parents, and the consequences of the persistence of abusive images on the internet

**DOI:** 10.1186/s12888-014-0295-7

**Published:** 2014-11-08

**Authors:** Ramón JL Lindauer, Sonja N Brilleslijper-Kater, Julia Diehle, Eva Verlinden, Arianne H Teeuw, Christel M Middeldorp, Wilco Tuinebreijer, Thekla F Bosschaart, Esther van Duin, Arnoud Verhoeff

**Affiliations:** Department of Child and Adolescent Psychiatry, Academic Medical Centre, Amsterdam, The Netherlands; De Bascule, Academic Center for Child and Adolescent Psychiatry, Amsterdam, The Netherlands; Department of Social Pediatrics, Emma Children’s Hospital, Academic Medical Centre, Amsterdam, The Netherlands; Department of Epidemiology & Health Promotion, Public Health Services, Amsterdam, The Netherlands; Department of Child and Adolescent Psychiatry, GGZ-InGeest/VU University Medical Center, Amsterdam, Netherlands; Department of Biological Psychology, VU University, Amsterdam, The Netherlands; Department of Sociology and Antropology, University of Amsterdam, Amsterdam, The Netherlands

**Keywords:** Sexual abuse, Infants, Very young children, Signs, Effects, Internet

## Abstract

**Background:**

Little research has been done on the signs of child sexual abuse (CSA) in infants and very young children, or on the consequences that such abuse - including the persistence of the abusive pornographic images on the internet - might have for the children and their parents. The effects of CSA can be severe, and a variety of risk- and protective factors, may influence those effects. CSA may affect the psychosocial-, emotional-, cognitive-, and physical development of children, their relationships with their parent(s), and the relations between parents. In the so called `the Amsterdam sexual abuse case’ (ASAC), infants and very young children were victimized by a day-care employee and most of the victims were boys. Research involving the children and their parents would enable recognition of the signs of CSA in very young children and understanding the consequences the abuse might have on the long term.

**Methods/design:**

The proposed research project consists of three components:

(I) An initial assessment to identify physical- or psychological signs of CSA in infants and very young children who are thought to have been sexually abused (n = 130);

(II) A cross-sequential longitudinal study of children who have experienced sexual abuse, or for whom there are strong suspicions;

(III) A qualitative study in which interviews are conducted with parents (n = 25) and with therapists treating children from the ASAC. Parents will be interviewed on the perceived condition of their child and family situation, their experiences with the service responses to the abuse, the effects of legal proceedings and media attention, and the impact of knowing that pornographic material has been disseminated on the internet. Therapists will be interviewed on their clinical experiences in treating children and parents.

The assessments will extend over a period of several years. The outcome measures will be symptoms of posttraumatic stress disorder (PTSD), dissociative symptoms, age-inappropriate sexual behaviors and knowledge, behavioral problems, attachment disturbances, the quality of parent-child interaction, parental PTSD, parental partner relation, and biological outcomes (BMI and DNA).

**Discussion:**

The ASAC-project would facilitate early detection of symptoms and prompt therapeutic intervention when CSA is suspected in very young children.

**Electronic supplementary material:**

The online version of this article (doi:10.1186/s12888-014-0295-7) contains supplementary material, which is available to authorized users.

## Background

### Problem definition

In late 2010, the Dutch Public Prosecution Service disclosed that a suspected 130 infants and very young children had been sexually abused by a male employee of an Amsterdam day care center. The episode had an enormous impact and drew considerable media attention at home and abroad. This article describes the longitudinal and qualitative study we have now started, which will monitor the abused children over a longer period of time.

International prevalence statistics indicate that 4 to 127 of 1,000 children or adolescents may be involved in child sexual abuse (CSA), depending on the type of informant and the child’s gender [1]-[7]. Prevalence figures are higher for girls than for boys, and 25% to 35% of the sexual abuse affects children younger than seven years of age [3].

CSA may produce short-, medium-, and long terms effects in children and parents [8]-[16]. In the short term, children may develop posttraumatic stress disorder (PTSD), problems with sexuality or sexual behavior, dissociative symptoms, psychosomatic complaints, emotional problems, anxieties, and sleep disturbances [17]-[31]. In the middle term (two to four years after abuse has ceased), parents report that one half of the children still have fears relating to the threat from the perpetrator(s) [32] and that one half still have serious PTSD symptoms [33]. In the long term, sexually abused children have been found to have a three times higher risk of developing mental health- and physical problems than non-abused children [34]-[39]. Research has shown that childhood traumatic and stressful experiences can lead to chronic health problems in later life, including cardiovascular disease, obesity, and diabetes [40],[41]. An Australian study (N = 2688) on the long-term effects of CSA demonstrated that about one quarter of the victims required mental health care over the course of a 43-year period, as compared to 8% of the control group [36].

If children who have experienced CSA develop serious health consequences it is partly connected to a range of risk- and protective factors. Risk factors involve the nature of the abuse (penetration makes it worse), the duration and frequency, the amount of violence used, and whether the perpetrator was a family member or relative [42]-[44]. Protective factors include parental support (if the abuse was outside the family).

A child’s temperament also influences whether or not symptoms develop. Although PTSD is always preceded by an environmental factor - the traumatic event - twin studies indicate that genetic factors have a 30%-40% influence on individual differences in susceptibility [45],[46]. This is comparable to the heritability of other disorders such as depression, yet for PTSD far less research has investigated the influence of genes on the onset of the disorder [47].

Excessive, repeated, or chronic stress in the initial years of life can deregulate the biological stress systems and induce epigenetic modifications - structural changes in DNA that make the DNA at that locus easier or more difficult to read. This affects the degree of expression of a gene, meaning that the risk of developing PTSD symptoms may be influenced not just by congenital genetic variation, but also by epigenetic modifications induced by the trauma [48].

Parents face the awesome task of dealing with their own reactions and feelings without distressing their child any further [49]. Common parental reactions are feelings of guilt, rage against the perpetrator, and fear that the abuse will be repeated in a different setting [50]. Parents have reported that being informed about the sexual abuse of their child, the police questioning, and the media attention are all highly stressful [33]. Parents may develop PTSD and depressive symptoms themselves [50]-[56]. This can adversely affect their relationships with their child, their partner, and the child’s siblings [57]-[59]. For young children, the quality of the attachment relationship with parents is important for their further development. Traumatic experiences, including sexual abuse, may alter the quality of the attachment relationship to parents; it may become insecure or even disorganized. Alternatively, parental care may have protective effects, with possible positive influences on biological processes such as epigenetic modifications or alterations in the functioning of the HPA axis [45],[46],[60]-[66]. Reduced coping capacities and parental concerns about issues such as sexual development (including homosexuality in abused boys) have been found to often raise new problems for families. In about 20% of cases, a negative spiral emerges that leads to prolonged psychopathology in the child and possibly in the parents.

If child pornography has been made the children are not only victims of the CSA they have suffered, but also of the fact that recorded images of the abuse persist [67]-[69]. The existence of such material can have traumatic effects on the child and the family as well giving the gross violation of their privacy [69]. If the abusive images have been placed on the internet, the children and their parents must live with the permanence of the images, since it is virtually impossible to ever completely remove them. The realization of that permanence, which often does not dawn until later, may trigger feelings of loss of control, helplessness, shame, and fear [69]. At present, little is known about what consequences the dissemination of pornographic material might have for the victimized children, or their parents. Only a few treatment programs exist that focus on these aspects of the victims’ experience [67].

In view of the probable serious consequences of CSA in the short-, medium-, and long terms, it is important to ensure early recognition of symptoms and prompt provision of support. Research literature about physical symptoms that might confirm suspicions of sexual abuse in pre-school children is very limited [70]-[73]. No valid diagnostic instruments are currently available. Barely any research has been conducted on the impact of CSA in the group of infants and very young children, usually this group is subsumed under a broader category labeled `younger than 6’ or `younger than 12’. Many children who are at risk of CSA are still in the preverbal phase which means that their expressive capabilities are limited [74]-[77]. Few specific details are known about the clinical picture for very young traumatized children. The limited research on physical injury in young children for whom a strong suspicion of CSA exists has found that just 4% to 14% of the children show signs of physical injury [70],[78]-[80].

Although CSA is common, it does rarely occur on the scale that it did in the Amsterdam sexual abuse case, committed by one chief perpetrator with one or two possible accomplices, and the severity of CSA is well documented by the police. CSA has grave consequences for the children involved, their parents, and their families. The quality of the parent-child relationship may be a protective factor. Systematic research on the effects that CSA has on very young children, and on boys in particular, is urgently needed to better understand the psychological and physical clinical presentations of such abuse and to identify the risk- and protective factors for psychopathology resulting from it. This knowledge will enable early recognition of problems and early intervention.

### Research questions and objectives

The purpose of the study is to systematically document the signs and symptoms of sexual abuse in infants and very young children and the short-, medium-, and long-term effects of the abuse, including the effects of the persistence of pornographic internet images, on the children and their parents. The study will examine the psychological, social, emotional, cognitive, and physical development, and developmental problems, of children, the psychological well-being of their parents, and the quality of interactions between parents and children, and between parents. The outcome measures will be symptoms of PTSD, dissociative symptoms, age-inappropriate sexual behavior and knowledge, behavioral problems, attachment disturbances in children, PTSD in parents, the quality of parent-child interaction, parental partner relation, and biological outcomes.

We will investigate the following types of questions:

• Child-focused questions:
  - ✓ What signs of sexual abuse of infants and very young children are apparent in the short term?
    - ■ What physical injuries do very young sexually abused children have?
    - ■ Does a relationship exist between the severity of the abuse (judged from information in police reports and criminal indictments) and the development of trauma symptoms in children?
    - ■ Does a relationship exist between parents’ own past experiences of abuse and the development of trauma symptoms in their children?
  - ✓ How does sexuality and sexual knowledge develop in children for whom early sexual abuse has been confirmed or strongly suspected?
  - ✓ What effects of early-age sexual abuse are apparent in the medium- and long terms, and to what extent are these influenced by biological factors?
  - ✓ What consequences does the persistence of pornographic internet images have for a child in medium and long terms?
• Parent-focused questions:
  - ✓ What psychological consequences are experienced by parents whose child has been abused?
  - ✓ What consequences does the persistence of pornographic Internet images have for parents in the medium and long terms?
  - ✓ What influence does the sexual abuse have on parental partner relationships?
• Influences of parent-child interaction and family environment:
  - ✓ What risk- and protective factors influence the development of symptoms in children and their parents for example social support, attachment problems, parental trauma history?
  - ✓ What influence does the quality of the parent-child relationship have on the development of symptoms in the children?
• Additional questions:
  - ✓ What are the experiences of parents who have, and who have not, informed their child about the sexual abuse the child experienced as an infant? If they have informed the child, how and at what age did they do so? What underlying motives do parents have for informing or not informing their children?
  - ✓ What clinical experiences are reported by therapists that treat children and parents for the effects of sexual abuse?

## Methods/Design

### Project organization

The following people and groups will be involved in the study: the project leaders, the project group, and a research advisory committee comprised of Dutch and international experts.

The project group will support the three project leaders (RJLL, SNB and AV) in all facets of the study. The project leaders will have charge of the day-to-day practical conduct of the study. The project leaders and the members of the project group will meet regularly. The research advisory committee will be informed once a year in writing and will make recommendations to the project group. Should the subject matter of the study require more consultation, meetings will be scheduled more frequently.

### Study design

#### Signs of sexual abuse in very young children

The initial assessment (T0) took place shortly after disclosure of the abuse case in December, 2010. At the Academic Medical Centre (AMC), five outpatient teams were appointed, composed of three types of professionals (pediatrician, parent adviser, and child psychologist or child development expert). Each team made systematic diagnoses of the physical and psychological effects of sexual abuse (confirmed or suspected) in children and their parents. A total of 130 children and their parents were examined; in 126 cases, parents authorized the use of anonymized data for research purposes. For 87 of the children, a criminal case file now exists in which sexual abuse has been demonstrated by investigators and/or acknowledged by the chief perpetrator.

### Longitudinal (cross-sequential) study

We will conduct a cross-sequential longitudinal study [81],[82] involving children who have experienced sexual abuse or for whom there are strong suspicions of abuse. A cross-sequential longitudinal design will enable us to systematically determine the effects of sexual abuse by combining three research techniques: (1) examining the same children at different ages (longitudinal study); (2) examining children of different ages at the same point in time (cross-sectional study); and (3) examining children of different ages at different points in time (cross-sequential study). The longitudinal study started in 2013 (T1). Some children who have experienced sexual abuse or for whom there are strong suspicions of abuse were not involved in the measurements of T0. However, their parents had contact with the Public Health Service during the second aftercare phase as part of the psychosocial support program for accidents and disasters. The Public Health Service provided this aftercare program with the goal to detect psychosocial complaints in traumatized victims. The research project will monitor children and their parents for several years (granted till 2017) to gather firm evidence on long-term effects.

### Qualitative study

In the qualitative study, parents and therapists will be interviewed by a psychologist/researcher. The focus of the parental interviews will be on the perceived condition of their child, their family situation, their experiences with the health and social services, the impact of legal procedures and media attention, the impact of the Internet dissemination of the child pornographic material, and the extent to which they recognize outcomes of the quantitative study. The focus of the interviews of the therapists will be on their experiences of treating children and parents involved in the abuse case.

### Study groups

#### Signs of sexual abuse in very young children

This group involves the children who have experienced sexual abuse or for whom there are strong suspicions of abuse (n = 126).

### Longitudinal study (cross-sectional)

The study group as known at the AMC (n = 126), comprised very young children from 4.5 months to 11.2 years of age (median age 3.9 years; 77 boys and 48 girls) who are confirmed or strongly suspected to have experienced sexual abuse (oral, vaginal, and/or anal). Confirmed sexual abuse is defined as abuse that has been demonstrated by police and/or affirmed by the chief perpetrator. Some children who are confirmed or strongly suspected to have experienced sexual abuse were not involved in the AMC study group (n = 126) but had contact with the Public Health Service during the aftercare program. Therefore we proceed on the assumption that parents of 150 children can be contacted for participation in this research project.

### Qualitative study

This group will involve a subgroup of participating parents. Following the first quantitative assessment in the longitudinal study (T1), parents will be recruited for qualitative interviews (n = 25; this number will suffice for the qualitative data, as we expect no new information from a larger sample). To recruit the interviewees, we will first compile a comprehensive list of parents who have expressed willingness to take part. From this list, we will select parents for the interviews. In this selection, it will be important to allow for gender, education, SES, ethnicity, and the age at which their child was abused. Therapists from various child and adolescent mental health care agencies will also be selected for interviews.

### Informed consent

The Medical Ethical Committee of the Academic Medical Centre, Amsterdam, the Netherlands approved on the research protocol.

At T0, we requested parents’ permission to use the collected data in anonymized form for publication in scholarly journals. After consultation with the medical director, we requested this consent orally and recorded it in the medical file. At the start of the longitudinal study (T1), parents will be requested to also provide written consent for T0.

Parents who took part in the T0 measure will be contacted by or in behalf of AMC-employees who were involved in the T0 measure. Parents will be asked if the Public Health Service may inform them about this longitudinal research project. Parents who are known to the Public Health Service from the second aftercare phase in 2011 will again be contacted by or in behalf of Public Health Service employees who were involved in the after care phase. Parents will be asked if they want to receive information about the longitudinal research project. Parents who want to receive information will be sent written information about the research study. If necessary, researchers will provide more detailed explanations in response to any parental questions. After a couple of days, written consent to participate will be requested. Separate written consent will be obtained from parents who are willing to take part in the study component in which children are also actively involved. Children who are older than 7 and who actively take part in the study will also be provided with information about the research. Children who are older than 11 will also be asked to sign an informed consent form. Children who are not informed about the sexual abuse will not be contacted for this research project. A separate written consent form will be requested from participants who also agree to biological testing.

The reason we have decided on the tiered informed consent procedure is to enable parents to take part in the project who do not wish to inform their child about the abuse. In those cases, we will contact the parents only, and will administer questionnaires and interviews to them about their child.

For examination of police files separate informed consent will be asked. Informed consent will also be asked for information about the received treatment and the effects.

### Assessments

In the present project we describe how we will monitor the children and their parents over a period of many years. This will depend on the ongoing consent of parents (and of children, if aged 12 or older). A number of assessments will take place: T0 (late 2010), T1 (2013), T2 (2014), T3 (2015), T4 (2016), T5 (2017), and T6 (2018).

#### Assessment instruments

***Background variables:*** demographic information, severity and type of sexual abuse, other stressors, family problems, legal procedures, and psychological treatment and its effects.

### Signs of sexual abuse in very young children

**The following assessment instruments were administered at T0 (late 2010):**

• Adams classification for genital abnormalities after sexual abuse [83]-[86]. Photographs of the external genitals were made in the “frog-leg” position (supine with knees bent and foot soles together) and the “polar bear” position (on knees and elbows). The pictures were taken in conformity with the guidelines for identifying physical signs of suspected sexual abuse and were assessed by a team of experts using the Adams classification. The interrater reliability of these assessments will be evaluated.
• Symptom score checklist compiled on the basis of a number of existing questionnaires for assessing mental health and well-being in children and parents, sexual abuse in parents’ past history, and family problems;
• The Sexual Knowledge Picture Instrument (SKPI; [87]) for estimating Children’s sexual development and knowledge;
• Information from police reports and indictments considering (nature and severity of the abuse); Public Health Services questionnaires (general questionnaire on demographic data; parental mental health problems such as depression; uptake of available treatment).

### Longitudinal study

#### Assessment instruments in the longitudinal study (Tand further)

Based on the type of consent that parents have given, we will create three different categories in the sample (assessment including parents only; assessment of parents and children; and assessment of parents and children, including biological testing). This will determine which questionnaires, interviews, observations, and biological tests will be used (see also Tables). All questionnaires will be delivered to parents via a digital link and the children full out the questionnaires at location. Interviews with parents and/or children will be conducted at the child and adolescent psychiatry of the AMC or, at an location of the Public Health Services Amsterdam. If parents prefer, interviews will be conducted at home. The primary outcome measures will be symptoms of PTSD, dissociative symptoms, behavioral problems, age-inappropriate sexual behavior and knowledge, attachment disturbances, the quality of parent-child interaction, PTSD in parents, and parental partner relation.

### Assessment instruments: questionnaires and interviews for parents

Psychological health and well-being of the child (questionnaires and interviews *administered to parents only*):

• PTSD/trauma symptoms:
  - ✓ CRIES-13, parent version (Children’s Revised Impact of Event Scale; [88]-[90]): a 13-item questionnaire to screen for PTSD symptoms in children. Internal consistency ranges from alpha 0.71 and 0.87. Convergent, divergent, and criterion validity are good [91].
  - ✓ DIPA (Diagnostic Infant and Preschool Assessment; [92],[93]). The DIPA is a semi-structured interview for PTSD and other anxiety disorders as well as mood, behavioral, reactive attachment, and sleep disorders in children up to age 7. The DIPA has been validated, is reliable, and has a test-retest reliability of kappa 0.53 [92].
  - ✓ ADIS-C (Anxiety Disorders Interview Structured for DSMIV-child version, [94]; Dutch version: [95]). This interview investigates PTSD and other anxiety disorders in children aged 8 to 17 years. The ADIS-C demonstrates good to excellent test-retest and inter-rater reliability [96],[97].
• Dissociative symptoms:
  - ✓ CDC (Child Dissociative Checklist; [98]; Dutch translation by [99]). The CDC is a list of 20 questions that provide indications of the presence of dissociative symptoms in children up to age 12. Test-retest reliability ranges from 0.57 to 0.92, and internal consistency from alpha 0.64 and 0.95 [98].
• Attachment disturbance symptoms:
  - ✓ AISI (Attachment Insecurity Screening Inventory, [100]) Attachment disturbances can be investigated with the AISI. This 20 items questionnaire will be administered to parents with children age 2 to 12 years.
  - ✓ GIH (Global Indicatielijst Hechting, [101]). The GIH is a 36 items questionnaire that investigates attachment disturbances in children older than 12.
• Sexual behavior (and problems):
  - ✓ CSBI (Child Sexual Behavior Inventory; [102]; Dutch translation by [103]). The CSBI is a questionnaire to screen for symptoms of inappropriate sexual behavior under the age of 12. Reliability is alpha 0.93, and validity and test-retest reliability are adequate [104].
• General psychological functioning and behavioral problems:
  - ✓ CBCL (Child Behavior Checklist; [105]). The CBCL assesses internalizing and externalizing problems in children aged 1,5 to 17 years. Reliability ranges from alpha 0.71 to 0.89, validity is adequate, and test-retest reliability is 0.90 [105].
• Quality of life:
  - ✓ Kidsscreen-10, parent version [106]. Kidscreen-10 is a 10-item questionnaire on quality of life in children aged 8 to 17 years. Internal consistency is alpha 0.78. [106].

Psychological health and well-being of the parent(s):

• PTSD/trauma symptoms/parental stress:
  - ✓ IES-R (Impact of Event Scale-Revised; [107]). The 22-item IES-R questionnaire is completed by parents to screen for PTSD in parents. Internal consistency ranges from alpha 0.79 to 0.90 and validity from 0.53 to 0.72 [107].
  - ✓ PERQ (Parent Emotional Reaction Questionnaire; [108]). The 15-item PERQ is completed by parents and assesses their emotional reactions to the sexual abuse of their child. Internal consistency is alpha 0.87 and test-retest reliability 0.90 [108].
• Parental relationship:
  - ✓ ECR (Experiences in Close Relationships; [109]; Dutch translation and validation by [110]). The ECR consists of 36 questions scored on a 7-point scale, testing two dimensions of attachment in adult partner relationships: (1) fear of rejection and abandonment by a partner; and (2) avoidance of intimacy. Internal consistency ranges from alpha 0.78 to 0.93, and test-retest reliability from 0.82 to 0.89 [111].

### Assessment instruments: questionnaires and interview for the child (if consent is given)

Psychological health and well-being of the child (the following questionnaires and interviews will be administered to the *child*, the children will have been informed about the sexual abuse):

• PTSD/trauma symptoms:
  - ✓ CRIES-13, child version (Children’s Revised Impact of Event Scale; [88]-[90]): a 13-item questionnaire to screen for PTSD in children aged 8 to 17 years. Internal consistency ranges from alpha .74 to .89. The test-retest reliability coefficient is .85 for the total score. Criterion validity is good [112].
  - ✓ CAPS-CA (Clinician-Administered PTSD Scale, child and adolescent version; [113]) is a semi-structured clinical interview for children aged 8 to 17 years that enables DSM-IV-TR PTSD diagnoses and severity scores of posttraumatic stress symptoms to be determined on a standardized basis. Internal consistency is alpha 0.83; validity and interrater reliability are good [114].
• CPTCI (Child Post-Traumatic Cognitions Inventory; [115]). matically screens for negative thoughts that traumatized children and adolescents, aged 8 to 18 years, might have about themselves or the world around them. Internal consistency, test-retest reliability, and convergent and discriminant validity are good [115].
• Dissociative symptoms:
  - ✓ ADES (Adolescent Dissociative Experience Scale; [116]). The ADES is a self-completion questionnaire that assesses dissociative symptoms in children age 12 years and older. Reliability and validity are good [116].
• General functioning and behavioral problems:
  - ✓ YSR (Youth Self-Report; [105]). The YSR assesses the behavioral and emotional functioning of adolescents (11 years and older), including internalizing and externalizing problems. Internal consistency ranges from 0.55 to 0.75, validity is good, and test-retest reliability is 0.88 [105].
• Quality of life:
  - ✓ Kidscreen-10, child version [106]. Kidscreen-10 is a 10-item questionnaire on quality of life in children aged 8 to 17 years. Internal consistency is alpha 0.82 and test-retest reliability 0.55 [106].

### Assessment instruments: observations child/parent (if consent is given)

Quality of parent-child interaction (observations):

• Strange situation procedure [117],[118]: During this separation and reunion procedure, the quality of the attachment relationship between children and their parent is assessed.
• A 25-minute video recording will be made of three play situations involving the child and a parent, with degree of control varying by situation: (1) free play, with parent following the child’s lead; (2) structured play, with parent taking more of a lead; and (3) tidying up the toys. The video recordings will then be coded using two coding systems: DPICS (Dyadic parent-child Interaction Coding System; [119]), and EAS (Emotional Availability Scales; [120]).

### Assessment instruments: biological measures (if consent is given)

• Weight and height will be measured and body mass index (BMI) calculated.
• The Children’s growth charts were requested from the Child Health Center.
• Genetic material (DNA) will be isolated from saliva, after which single nucleotide polymorphisms (SNPs) and methylation status will be determined on a genome-wide basis. This study will examine whether SNPs can be identified that are associated with posttraumatic stress symptoms. We will also analyze whether methylation status affects the degree of symptomatology in the group of abused children [45],[46].

### Qualitative study

Individual interviews will be conducted with 25 parents, guided by the following topics: perceived condition of the child and the family situation, experiences with health and social services in the preceding period, experiences with legal procedures, experiences with media attention, viewpoints on the impact of child pornography, in particular on the internet. The interviews will have an open structure. Interviewees will be invited to tell their own story. The subjects in the topic guide will be raised by the interviewer if they fail to come up naturally. The interviewer will probe for respondents’ experiences and also experiences of the therapists who treated these children.

### Outcome measures

See Tables 1, 2, and 3 for the outcome measures.

**Table 1:** **Consent for contact with parents only (questionnaires are adult/parent versions)**

Assessment instrument	Questionnaire or interview	Construct	Standardized/validated	Age of the child
CRIES	Questionnaire	PTSD symptoms	yes, in USA and in the Netherlands	2-18 years
DIPA or ADIS-C	Interview	diagnosis and symptoms of PTSD, other anxiety disorders, and mood, behavioral, reactive attachment, and sleep disorders	yes, in USA and a Dutch study in progress	2-18 years
CDC	Questionnaire	symptoms of dissociation	yes, in USA	5-14 years
AISI or GIH	Questionnaire	symptoms of inhibited and disinhibited attachment	yes, in the Netherlands	2-18 years
CSBI	Questionnaire	symptoms of inappropriate sexual behavior	yes, in USA Dutch study in progress	2-12 years
CBCL	Questionnaire	internalizing and externalizing symptoms	yes, internationally	1.5-5 years and 6-18 years
Kidscreen-10	Questionnaire	quality of life	yes, internationally	8-18 years
IES-R	Questionnaire	parental PTSD symptoms	yes, internationally	parents
PERQ	Questionnaire	parental emotional reactions to sexual abuse of child	no	parents
ECR	Questionnaire	attachment in adult partner relationships	yes, in USA and in the Netherlands	parents

**Table 2:** **Questionnaires/interviews child**

Assessment instrument	Questionnaire or interview	Construct	Standardized/validated	Age of the child
CRIES	questionnaire	PTSD symptoms	yes, in USA and in the Netherlands	8-18 years
CAPS-CA	interview	PTSD diagnosis and symptoms	yes, in USA and in the Netherlands	8-18 years
InADES	questionnaire	symptoms of dissociation	yes, in USA and Turkey	12-20 years
YSR	questionnaire	internalizing and externalizing symptoms	yes, internationally	11-18 years
CPTCI	questionnaire	negative cognitions about oneself and the world	yes, Dutch study in completion	8-18 years
Kidscreen-10	questionnaire	quality of life	yes, internationally	8-18 years

**Table 3:** **Observation of parent-child play interaction**

	Type	Construct	Standardized/ validated	Age of the child
**Strange situation procedure**	Observation of child-parent interaction	Assessment of quality of attachment	yes, internationally	< 8 years
**DPICS/EAS**	observation of parent-child play interaction	assessment of parenting and quality of interaction	yes, internationally	< 8 years

### Time line and sample selection

Parents were contacted by the AMC or the Public Health Service Amsterdam and invited to take part in the study. Researchers will be employed who will receive training in conducting interviews and observations, and in scoring the observations, from the Child and Adolescent Psychiatry Research Group at the AMC.

The T1 assessments took place in mid-2013, and the data obtained will be processed, analyzed, and reported later. In the first half of 2014, preparations will be made for the T2 assessments. This working procedure will enable us to perform, process, analyze, and report on the previous year’s assessment as well as preparing the subsequent assessments.

### Statistical analysis

Categorical data will be summarized as frequencies and percentages, continuous data will be summarized by means and standard deviations or median and interquartile scores, and if appropriate Linear mixed model analysis will be used to investigate the course of the outcomes of interest (e.g. PTSD dissociative symptoms) and to examine and test the effects of the various child- and abuse characteristics on the outcomes. Longitudinal models for data assembled at T1 and further will have a random intercept to account for individual differences at baseline, fixed effects regression coefficients for each of the follow-up occasions after baseline while controlling for possible other confounding variables. The appropriate covariance structure for the longitudinal models and possible interaction effects will be assessed using log-likelihood statistics. Growth curves will be examined by plotting data and when appropriate we will include polynomials reflecting the observed curves. Cross-sectional comparisons at different time-points will also made using linear mixed model or ordinary multivariable regression analysis to examine the effects of child/parent and abuse characteristics on the outcomes.

### Confidentiality of study data

The data for this study will be collected by researchers from the Department of Child and Adolescent Psychiatry at the AMC and the Department of Epidemiology and Health Promotion, at the Public Health Service in Amsterdam. The Clinical Research Unit of the AMC will be asked to build and manage the database for this project. The data will be stored in coded form by assigning a number to each file, questionnaire, and scoring sheet. This will prevent unauthorized persons from tracing which file belongs to which child or parents. Study results will also be published in anonymized form.

## Discussion

The ASAC-study will be the first longitudinal study to systematically investigate signs and symptoms of sexual abuse in infants and very young children, and on boys in particular. These effects of the abuse will be measured at the short-, medium-, and long-term. The effects of the persistence of pornographic internet images, on the children and their parents, will also be investigated. Although CSA is common, it rarely occurs on the scale that it did in the Amsterdam sexual abuse case, committed by one chief perpetrator with one or two possible accomplices, and the severity of CSA is well documented by the police. Systematic research on the effects that CSA has on very young children is urgently needed to better understand the psychological and physical clinical presentations of such abuse and to identify the risk- and protective factors for psychopathology resulting from it. It is expected that CSA has effects on the child, parents, child-interaction and parental partner relation. This knowledge will enable early recognition of problems and prompt therapeutic intervention when CSA is suspected in very young children.

## Authors' contributions

RL, SB, JD, EV, AT and AV wrote the current manuscript. RL, SB and AV are the project leaders. AT, SB, and TB are responsible for the first assessment in 2010. JD and EV are responsible for the assessment in 2013. All authors read and approved the final manuscript.

## Authors’ original submitted files for images

Below are the links to the authors’ original submitted files for images. Authors’ original file for figure 1 Authors’ original file for figure 2 Authors’ original file for figure 3
