# Supplementary material for: The Amsterdam Sexual Abuse Case (ASAC)-study in day care centers: longitudinal effects of sexual abuse on infants and very young children and their parents, and the consequences of the persistence of abusive images on the internet
Source: BMC Psychiatry. 2014 Nov 8;14:295. doi: 10.1186/s12888-014-0295-7 (PMC4240883; doi:10.1186/s12888-014-0295-7)
Supplement: Supplementary file 1 — Authors’ original file for figure 1 [file 12888_2014_295_MOESM1_ESM.docx]

**Table 1a.** Consent for contact with parents only (questionnaires are adult/parent versions)

| Assessment instrument | Questionnaire or interview | Construct | Standardized/validated | Age of the child |
| --- | --- | --- | --- | --- |
| CRIES | Questionnaire | PTSD symptoms | yes, in USA and  in the Netherlands | 2-18 years |
| DIPA or ADIS-C | Interview | diagnosis and symptoms of PTSD, other anxiety disorders, and mood, behavioral, reactive attachment, and sleep disorders | yes, in USA  Dutch study in progress | 2-18 years |
| CDC | Questionnaire | symptoms of dissociation | yes, in USA | 5-14 years |
| AISI or GIH | Questionnaire | symptoms of inhibited and disinhibited attachment | yes, in the Netherlands | 2-18 years |
| CSBI | Questionnaire | symptoms of inappropriate sexual behavior | yes, in USA  Dutch study in progress | 2-12 years |
| CBCL | Questionnaire | internalizing and externalizing symptoms | yes, internationally | 1.5-5 years and 6-18 years |
| Kidscreen-10 | Questionnaire | quality of life | yes, internationally | 8-18 years |
| IES-R | Questionnaire | parental PTSD symptoms | yes, internationally | parents |
| PERQ | Questionnaire | parental emotional reactions to sexual abuse of child | no | parents |
| ECR | Questionnaire | attachment in adult partner relationships | yes, in USA and in the Netherlands | parents |
