# Supplementary material for: The Amsterdam Sexual Abuse Case (ASAC)-study in day care centers: longitudinal effects of sexual abuse on infants and very young children and their parents, and the consequences of the persistence of abusive images on the internet
Source: BMC Psychiatry. 2014 Nov 8;14:295. doi: 10.1186/s12888-014-0295-7 (PMC4240883; doi:10.1186/s12888-014-0295-7)
Supplement: Supplementary file 2 — Authors’ original file for figure 2 [file 12888_2014_295_MOESM2_ESM.docx]

**Table 1b.** Questionnaires/interviews child

| Assessment instrument | Questionnaire or interview | Construct | Standardized/validated | Age of the child |
| --- | --- | --- | --- | --- |
| CRIES | questionnaire | PTSD symptoms | yes, in USA  and in the Netherlands | 8-18 years |
| CAPS-CA | interview | PTSD diagnosis and symptoms | yes, in USA  and in the Netherlands | 8-18 years |
| InADES | questionnaire | symptoms of dissociation | yes, in USA and Turkey | 12-20 years |
| YSR | questionnaire | internalizing and externalizing symptoms | yes, internationally | 11-18 years |
| CPTCI | questionnaire | negative cognitions about oneself and the world | yes,  Dutch study in completion | 8-18 years |
| Kidscreen-10 | questionnaire | quality of life | yes, internationally | 8-18 years |
