# Supplementary material for: The Amsterdam Sexual Abuse Case (ASAC)-study in day care centers: longitudinal effects of sexual abuse on infants and very young children and their parents, and the consequences of the persistence of abusive images on the internet
Source: BMC Psychiatry. 2014 Nov 8;14:295. doi: 10.1186/s12888-014-0295-7 (PMC4240883; doi:10.1186/s12888-014-0295-7)
Supplement: Supplementary file 3 — Authors’ original file for figure 3 [file 12888_2014_295_MOESM3_ESM.docx]

**Table 1c.** Observation of parent-child play interaction

|  | **Type** | **Construct** | **Standardized/ validated** | **Age of the child** |
| --- | --- | --- | --- | --- |
| **Strange Situation Procedure** | Observation of child-parent interaction | Assessment of quality of attachment | yes, internationally | < 8 years |
| **DPICS/EAS** | observation of parent-child play interaction | assessment of parenting and quality of interaction | yes, internationally | < 8 years |
